# Utilizing an Educational Intervention to Enhance Influenza Vaccine Literacy and Acceptance Among Minoritized Adults in Southern Californian Vulnerable Communities in the Post-COVID-19 Era

**DOI:** 10.3390/idr17020018

**Published:** 2025-02-26

**Authors:** Jacinda C. Abdul-Mutakabbir, Raheem Abdul-Mutakabbir, Samuel J. Casey

**Affiliations:** 1 Division of Clinical Pharmacy, Skaggs School of Pharmacy and Pharmaceutical Sciences, University of California San Diego, La Jolla, CA 92093, USA; 2 Division of Black Diaspora and African American Studies, University of California San Diego, La Jolla, CA 92093, USA; 3 Eastern Michigan University, Ypsilanti, MI 48197, USA; rabdul@emich.edu; 4 Congregations Organized for Prophetic Engagement, San Bernardino, CA 92374, USA; scasey@copesite.org

**Keywords:** vaccine literacy, vaccine acceptance, health equity, vaccine equity

## Abstract

Background/Objectives: Since the COVID-19 pandemic began, vaccination rates for preventable diseases, including influenza, have significantly dropped among racially and ethnically minoritized (REM) individuals in the United States. This study explored the effects of a community-based educational intervention designed to improve influenza vaccine literacy and acceptance among vulnerable REM individuals. Methods: The intervention included four 45 min interactive educational sessions on the influenza vaccine. The session attendees (18+) were invited to participate in a pre-/post-intervention study where an anonymous survey measured their post-COVID-19 pandemic attitudes, knowledge, and behaviors regarding the influenza virus and vaccine. To assess the effect of the intervention on vaccine literacy, we used a Mann–Whitney U test to test for differences between the pre-/post-intervention survey responses to seven knowledge-based questions. Descriptive statistics were employed to assess the impact of intervention on vaccine acceptance. Results: A total of 116 participants completed the pre-intervention survey, and 90 (78%) completed the post-intervention survey. All (100%) identified as REM, and 99% lived in highly vulnerable areas. Only 43% believed they were at risk for viral infection before the intervention, but 60% said the intervention helped them reassess their risk. We found significant differences in vaccine literacy when comparing the pre-/post-intervention survey responses, particularly regarding guideline-based vaccine recommendations (*p* < 0.05). Before the intervention, 65% of the participants indicated a high likelihood of receiving the influenza vaccine. In contrast, after the intervention, 81% of respondents indicated a high likelihood of being vaccinated, and 72% indicated that they were “extremely likely” to receive the immunization. Conclusions: Community-based educational interventions can have a positive impact on influenza vaccine literacy and acceptance among vulnerable REM populations in the post-COVID-19 era.

## 1. Introduction

The coronavirus disease-19 (COVID-19) pandemic has brought attention to the challenges associated with low vaccination rates among vulnerable groups, particularly racially and ethnically minoritized (REM) individuals, which includes American Indian and Alaska Native, Black, Hispanic/Latino, and Native Hawaiian/Pacific Islander individuals [1,2,3,4]. Specifically, during the peak of the pandemic, REM individuals that identified as either Black, Hispanic/Latino, or American Indian and Alaska Native were approximately twice as likely to die from COVID-19 compared with non-Hispanic White individuals [5,6]. Despite the significant impact of the disease on these REM groups, fewer than 60% of Black, Hispanic/Latino, or American Indian and Alaska Native individuals in the US have completed the recommended full vaccination series for COVID-19, and an even smaller percentage has received the recommended updated booster immunizations [7,8].

The COVID-19 pandemic has also been met with the decreased uptake of vaccines for other preventable diseases, including influenza [9,10]. In 2021, hospitalization rates for influenza were nearly 80% higher among REM adults compared with their non-Hispanic White counterparts [11]. Alarmingly, despite rising infection rates and related health complications, REM individuals were 50% less likely to receive vaccinations during the 2021–2022 influenza season than non-REM individuals [11,12]. As a result, vaccination rates reached some of the lowest numbers seen in the last decade [11,12]. This troubling trend is worsened by the fact that REM individuals are disproportionately affected by chronic diseases, such as diabetes, hypertension, and chronic obstructive pulmonary disease (COPD), which can lead to worse outcomes in infectious diseases [13,14,15,16].

There is limited data on the barriers to influenza vaccine uptake among REM individuals in the post-COVID-19 pandemic era. However, existing research points to inequities in social determinants of health (SDoHs) as significant contributors to disparities in vaccination rates [1,17]. Key factors include a lower socioeconomic status (SES), inadequate education, and limited access to healthcare [17,18,19]. In the US, vulnerable communities characterized by a low SES often have a higher concentration of REM individuals, a situation exacerbated by systemic housing policies that have led to residential segregation [18,20]. These low-SES neighborhoods typically offer fewer educational opportunities and have a larger number of individuals who do not complete high school [21,22]. This lack of education negatively impacts health outcomes, as studies indicate that REM individuals are more likely to have limited health literacy (LHL) [23]. Specifically, research shows that 58% of Black individuals and 41% of Hispanic/Latino individuals have basic or below-basic health literacy [23,24].

A downstream effect of overall low health literacy (LHL) is a lack of vaccine literacy. Lorini et al. describe vaccine literacy to be strongly associated with health literacy [25,26,27]. The authors further define vaccine literacy as people’s and communities’ knowledge, motivation, and competencies to access, understand, and critically appraise and apply information about immunization, vaccines, vaccination programs, and organizational processes to access vaccination. This includes the ability to navigate the health system and make informed decisions about vaccines for themselves, their families, and their communities, as well as to understand the larger global impact of vaccines concerning population health.

Of note, a study conducted in the US found that REM individuals were 13% more likely than White individuals to report being unaware of recommendations for receiving the influenza vaccine [28,29]. This lack of vaccine-related information has been linked to low immunization acceptance across REM groups [29,30]. Furthermore, a deficiency in understanding how vaccines work, as well as their safety and effectiveness, has also been associated with a low acceptance and uptake of the influenza vaccine among REM individuals [29,31,32]. These gaps in vaccine literacy may contribute to the perception of influenza as a mild illness that does not require preventative care, as noted in previous studies that examined barriers to vaccine uptake within REM populations [29,33,34,35].

Vulnerable communities also encounter profound challenges related to healthcare access, which may hinder immunization [36]. During the COVID-19 pandemic, research showed that REM individuals residing in areas with high social vulnerability scores had lower vaccination rates than those with lower vulnerability scores [37]. This disparity in vaccine acceptance and uptake is potentially due to the prevalence of “healthcare deserts” and provider shortages within these communities [38]. The lack of access to healthcare providers restricts the availability of trustworthy and culturally relevant vaccine information that can help individuals make informed decisions about vaccinations [39].

Community-based interventions emerged as effective strategies to rebuild trust and address vaccine literacy and acceptance limitations recognized across REM communities [2,40,41]. In 2021, a community–academic partnership was developed between a faith-based organization (FBO) and an academician from a local university to address the low COVID-19 vaccination rates across vulnerable REM communities in San Bernardino County (SBC), located in Southern California [42,43,44]. The community–academic partnership yielded the creation of a community-based educational intervention that consisted of the academician (a pharmacist by training) providing a 45 min PowerPoint presentation on the COVID-19 virus and vaccine at churches located in vulnerable REM SBC communities to enhance knowledge and encourage acceptance of COVID-19 immunization [42,45]. Research that assessed the baseline and post-changes following the educational sessions revealed increased COVID-19-related vaccine literacy among the participants, particularly related to their knowledge of COVID-19 viral risks and the intricacies associated with vaccine development and availability [45]. The research also revealed a 12% increase in vaccine acceptance among community members after attending these educational sessions [45].

Despite the low cost and widespread ability of influenza vaccines through pharmacies or healthcare providers, low uptake rates were reported across vulnerable REM groups across SBC and surrounding counties following the COVID-19 pandemic [46,47]. To address this, the community–academic team developed a community-based intervention to gather insights on the post-COVID-19 pandemic perspectives of vulnerable SBC residents regarding the influenza virus and vaccine. We also provided tailored education on the influenza virus to promote confidence in the vaccinations. Here, we describe the impact of our educational intervention on the attitudes, literacy, and acceptance of the influenza vaccine among these vulnerable REM individuals.

## 2. Materials and Methods

### 2.1. Study Setting and Design

The intervention, a 45 min vaccine education session, took place from September 2023 to December 2023, a time of heightened viral transmission in the Northern Hemisphere. First, a community–academic team, comprised of a project member from the FBO and an academician, collaboratively identified four church locations in highly vulnerable SBC communities, majorly populated by REM groups, to participate in the intervention. To determine these locations, the Centers for Disease Control and Prevention (CDC) Social Vulnerability Index (SVI) was utilized [4]. The CDC SVI uses census tracts (zip code level data) to determine community-level vulnerability. The index uses four themes: socioeconomic status, household characteristics, racial and ethnic minority status, and housing type and transportation, and stratifies social vulnerability into four categories: low, low-medium, medium-high, and high [4].

After identifying each church, a project manager from the FBO reached out to the faith leader of the ministry. During this initial contact, the academician provided information about the proposed intervention, along with a memorandum of understanding (MOU) that outlined the tasks to be completed. The churches were incentivized with USD 1000 to recruit at least 15 individuals from their community, aged 18 or older, to participate in the 45 min vaccine education session.

The 45 min interactive PowerPoint education session, led by the academician, focused on the factors influencing influenza vaccine acceptance and uptake among REM individuals. The PowerPoint presentation began by highlighting the risks associated with influenza and the significant burden the disease poses on REM groups. The presentation then covered the mechanisms of action of the influenza vaccine, the steps involved in its development, the benefits of vaccination, and guideline-based recommendations for immunization. The presentation concluded with detailed information on influenza vaccine accessibility and affordability options. For completion purposes, the educational session also included updated information on the risks of COVID-19 and respiratory syncytial virus (RSV) infections, emphasizing the importance of immunization against these viral diseases. All educational materials were developed using peer-reviewed, evidence-based literature and information from the CDC website, presented in plain language for the attendees.

The community members were incentivized with a USD 20 gift card to a local grocer to participate in a pre-/post-intervention study, which required them to complete a survey directly before and directly following the vaccine education session.

### 2.2. Pre-/Post-Intervention Survey Study

Upon review of the literature, the community–academic team was unable to identify a survey tool optimized to assess this study’s aims. Therefore, we developed the survey instruments—using existing literature—to collect information on the attitudes, knowledge, and behaviors of the participants at the baseline and following the intervention [29,32,35,48]. The survey tools were reviewed and validated using face validity by experts in the field. The survey tools were also piloted among REM individuals in a vulnerable SBC community one month (August 2023) before the intervention commenced. These individuals were not included in the final analysis of the results, and the Cronbach alpha value obtained from the pilot assessment was 0.71.

The 24-item pre-intervention survey consisted of three sections, and “flu” was used instead of influenza to prioritize the use of plain language throughout the survey. The first section was focused on collecting information about the participants’ perceptions of viral risks, barriers to receiving the vaccine (such as accessibility and the risk of adverse effects), perceived benefits of vaccination, and the perceived prevalence of vaccine uptake within the US and their respective communities. The health belief model was used to guide the development of this section, as it was used previously to explain and predict individual changes in behaviors related to health promotion and influenza vaccine uptake [49,50]. The second section included seven true or false statements that tested knowledge about the influenza virus, the vaccine, and guideline-based recommendations. The final section contained a single vaccine-acceptance-related question that inquired about the participants’ intent to get vaccinated before receiving education. For the questions in the first and third sections, the respondents provided their answers using Likert scales. The wording of the Likert scale response options was adapted to ensure maximum readability. The pre-intervention survey questions are shown in Supplemental Document S1. Only the individuals who completed the pre-intervention survey were allowed to complete the post-intervention survey.

The 19-item anonymized post-intervention survey was also divided into three sections. The first section evaluated the effectiveness of the education session in influencing participants’ attitudes and perceptions about the influenza virus and vaccine. This section also included a vaccine-acceptance-related question that assessed the level of importance that the participants placed on receiving the influenza vaccine after receiving the tailored education. The second section repeated the seven true-or-false knowledge-based statements from the pre-intervention survey. This repetition aimed to assess the impact of the education session on the participants’ influenza vaccine literacy. The third and final section of the post-intervention survey included two vaccine-acceptance-related questions. The first question in the third section evaluated the participants’ intent to be vaccinated, and the second evaluated their willingness to recommend the vaccine to a family or friend after participating in the educational session. The post-intervention survey questions are shown in Supplemental Document S2. Like the pre-intervention survey, the respondents answered the questions in the first and third sections using a Likert scale.

### 2.3. Statistical Analysis

The sample size estimation analysis concluded that for a moderate effect size (d = 0.50), α = 0.05, and 80% power, a minimum of 35 participants for both surveys would be required. The analysis was conducted using R version 4.4.2 [51].

Paper-based surveys were used during the intervention for the convenience of the participating community members. Directly following the intervention, trained individuals inputted the data into Qualtrics for analysis. We used counts and percentages to summarize the demographic data, which include details on race, ethnicity, gender, age, and SVI collected from the pre-intervention survey. Since only the individuals who completed the pre-intervention survey were eligible to take the post-intervention survey, we did not collect demographic data for the latter. Demographic data are shown in Table 1. Alongside the demographic information obtained from the pre-intervention survey, we also report the percentages of the participants’ responses to questions about their perceptions regarding the following topics: the risks of contracting influenza, challenges to vaccination, the benefits of vaccination, and community-level influenza vaccine uptake. Additionally, we report the counts and percentages to the pre-intervention survey vaccine-acceptance-related question, which inquired about the participants’ baseline intent to be vaccinated. All counts and percentages for the responses to the pre-intervention survey questions are shown in Table 2.

**Figure 1:**
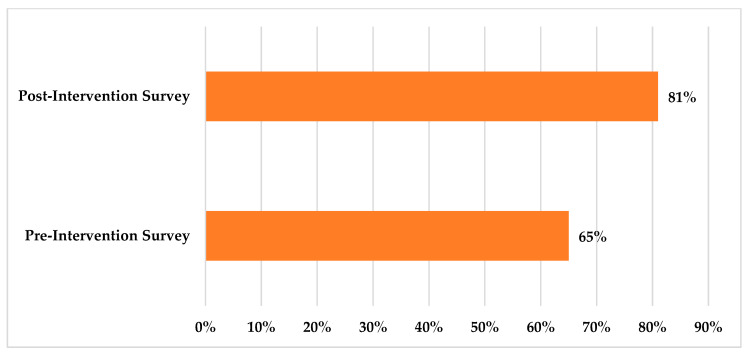
Percentage of individuals with a high likelihood of receiving the influenza vaccine (pre-intervention survey vs. post-intervention survey) (measurement of vaccine acceptance). Shown in Figure 1 is a comparison of the percentage of individuals that indicated a high likelihood of receiving the influenza vaccine in the pre- and post-intervention surveys. For the pre-intervention surveys, a high likelihood is shown by the additive percentage of those that selected “likely”, “very likely”, and “extremely likely” for the “intent to be vaccinated” pre-intervention survey question. For the post-intervention surveys a high likelihood is shown by the individuals who selected “4” or “5” on the Likert scale for the “intent to be vaccinated” post-intervention survey question.

From the post-intervention data, we present the average scores to questions related to the reflecting participants’ attitudes about the effectiveness of the education session in reforming their attitudes about the influenza virus and the vaccine. We also provide the percentage of the individuals who responded with a “5” or “extremely effective”. Additionally, we use percentages to summarize the participants’ reported trust in the information provided in the education session. Furthermore, we report the percentages of the post-intervention survey vaccine-acceptance-related questions that inquired about the participants’ intent to be vaccinated and to recommend the vaccine to others after receiving education. We also report the counts and percentages of the individuals who responded with “4” and “5”, indicating a high likelihood. A bar graph comparison of the percentages of individuals who indicated a high likelihood of vaccination in the pre- and post-intervention surveys is presented in Figure 1. The counts and percentages for the responses to the post-intervention survey questions are shown in Table 3.

**Figure 2:**
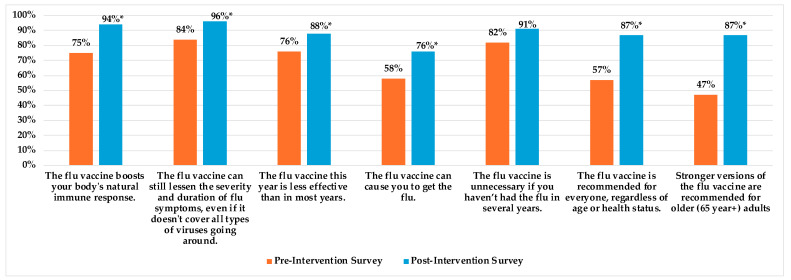
Percentage of correct responses to the knowledge-based true or false statements (pre-intervention survey vs. post-intervention survey) (measurement of vaccine literacy shift). Shown in Figure 2 is a comparison of the percentages of correct responses to the seven true or false influenza knowledge-based statements included in the pre- and post-intervention surveys. “Flu” was used instead of influenza to prioritize the use of plain language throughout the survey. * denotes a statistically significant difference (*p* < 0.05).

To assess the impact of the intervention on vaccine literacy, we compared the correct responses to the seven true or false knowledge-based statements in the second section of the pre-/post-intervention surveys. Mann–Whitney U tests were performed to test for statistical differences between the pre- and post-intervention survey correct responses using non-parametric ranking methods. Figure 2 shows a graphical representation of the comparison of the percentages of correct responses to the seven knowledge-based statements included in the pre-/post-intervention survey. Table S1 shows the counts and percentages of the percentages of correct responses to the seven knowledge-based statements included in the pre-/post-intervention survey. All statistical analysis was performed in R version 4.4.2. and statistical significance was determined as a *p*-value less than 0.05 (*p* < 0.05) [51].

## 3. Results

### 3.1. Demographics of Pre-Intervention Survey Participants

A total of 116 participants completed the pre-intervention survey, while 90 (78%) participants completed the post-intervention survey. Among the respondents of the pre-intervention survey, the majority identified as non-Hispanic Black or African American (99/116, 85%), followed by smaller proportions identifying as Hispanic/Latino(a) (9/116, 8%), American Indian or Alaskan Native (1/116, 1%), two or more races (4/116, 3%), and other/unlisted (3/116, 3%). The largest age group represented was those aged 65 or older (51/116, 44%), followed by participants aged 55 to 64 (25/116, 22%). Women comprised 70/116 (60%) of the participants, while men accounted for 46/116 (40%). Nearly all respondents (115/116, 99%) were classified as having a high level of social vulnerability. All demographic data on the baseline survey participants can be found in Table 1.

### 3.2. Pre-Intervention Perceptions of the Influenza Virus and Vaccine

The participants’ perspectives before the intervention revealed varying levels of knowledge regarding the influenza virus and the vaccine. In response to survey questions about their perceived risk of influenza infection, 47/116 (41%) participants believed it was “unlikely” that they would contract the flu, while 32/116 (28%) thought it was “likely”. Most participants anticipated experiencing “mild” (75/116, 65%) or “negligible” (10/116, 9%) symptoms if infected. Additionally, over half (64/116, 55%) expressed that they were “not worried at all” about severe outcomes, such as hospitalization. Regarding the perceived benefits of the influenza vaccine, 54/116 (47%) rated it as “very effective” at reducing complications, such as hospitalization. Furthermore, 48/116 (41%) believed it was “very effective” at reducing symptom severity. When examining perceived barriers to vaccination, most participants found the vaccine “very affordable” (77/116, 66%) and considered the vaccination process “very convenient” (82/116, 71%). However, 20/116 (17%) perceived a “very likely” chance of experiencing side effects. In terms of participants’ perceptions of community influenza vaccine uptake, 65/116 (58%) thought that “many” people in the US received the vaccine annually, but only 52/116 (45%) believed this was true for their local community. When asked about their intent to receive the influenza vaccine if a convenient and easily accessible location were available, 76/116 participants (65%) indicated a high likelihood (choosing “likely”, “very likely”, or “extremely” on the Likert scale) of getting vaccinated. All counts and percentages of responses to questions that assessed the baseline perceptions of the influenza virus and vaccine can be found in Table 2.

### 3.3. Post-Intervention Survey Responses on the Impact of the Intervention on Influenza Virus and Vaccine Perceptions

The post-intervention survey results indicate a major shift in the participants’ attitudes following the educational session. Specifically, when rating the effectiveness of the intervention on a scale of 1 to 5—where one means “not effective” and five means “extremely effective”—the participants gave high scores in several key areas. They rated the session highly for increasing their knowledge (average score: 4.84), helping them trust vaccine safety (average score: 4.77), prompting them to reevaluate their own risk of contracting the flu this season (average score: 4.65), aiding their understanding of the benefits of the flu vaccine (average score: 4.85), and addressing misconceptions about the vaccine (average score: 4.67). Awareness of influenza’s severity also showed substantial change, with an average score of 4.82; additionally, 60% of participants reported reevaluating their risk of infection.

Regarding the trustworthiness of the information provided in the educational session, when asked the following question: “How much do you trust the information provided in the presentation about flu vaccines?”, the participants gave a high average score of 4.87, indicating considerable trust in the educational materials. The average scoring of each question and the counts and percentage of individuals that responded with a “5” (the highest scoring option) are shown in Table 3.

### 3.4. Post-Intervention Survey Responses to Vaccine-Acceptance-Related Questions

In the post-intervention survey, three questions related to vaccine acceptance were posed. When participants were asked, “How important do you believe it is for you to get the flu vaccine?”, a substantial majority (74/90, 82%) selected “5” on the Likert scale, indicating that they felt receiving the vaccine was “extremely important” (average score: 4.6). Additionally, 84/90 participants (93%) expressed a high likelihood of recommending the vaccine to a family member or friend, selecting either “4” or “5” on the Likert scale (average score: 4.7). Furthermore, 73/90 respondents (81%) indicated they had a high likelihood of receiving the vaccine (selection of “4” or “5”), with an average score of 4.5, and 65/90 participants (72%) stated they were “extremely likely” to receive the vaccine by choosing “5” on the Likert scale. Both scoring percentages were higher than those reported in the pre-intervention survey. The average scores for each of these questions, as well as the counts and percentages of individuals who selected “5” (the highest scoring option), are detailed in Table 3. For the two questions that measured the intent to get vaccinated and the intent to recommend the vaccine to others, we also display the counts and percentages of individuals who responded with “4” and “5”, indicating a high likelihood. A bar graph comparison of the percentages of individuals who indicated a high likelihood of vaccination in the pre- and post-intervention surveys is presented in Figure 1.

### 3.5. Pre-Intervention Survey vs. Post-Intervention Survey Responses to the Seven Knowledge-Based True or False Statements

When comparing the correct responses to the seven knowledge-based true or false statements included in both the pre-and post-intervention surveys, we saw a significant shift in vaccine literacy from the baseline. Notably, awareness that the influenza vaccine enhanced the immune response changed from 75% before the intervention to 94% afterward (*p*-value < 0.05). Understanding that stronger influenza vaccines are recommended for older adults rose from 47% to 87% (*p*-value < 0.05). Additionally, fewer participants believed the vaccine caused influenza, where correct responses increased from 58% to 76% (*p*-value < 0.05). Knowledge also improved from the baseline concerning the statement that the flu vaccine can still reduce the severity and duration of influenza symptoms, even if it does not cover all types of circulating viruses; the correct responses changed from 84% to 96% (*p*-value < 0.05). Regarding perceptions about effectiveness, the percentage of respondents that indicated that this year’s flu vaccine is less effective than in most years changed from 76% in the pre-intervention survey to 88% in the post-intervention survey (*p*-value < 0.05). Furthermore, awareness that the flu vaccine is recommended for everyone, regardless of age or health status, improved from 57% in the baseline survey to 87% (*p*-value < 0.05) in the post-intervention survey. While we did note a shift in the number of correct responses to the statement “The flu vaccine is unnecessary if you haven’t had the flu in several years” (82% vs. 91%, in the pre-and post-intervention surveys, respectively). This shift was not statistically significant, at a *p*-value of 0.06 (*p* > 0.05). A bar graph comparison of the percentages of individuals who responded correctly to the seven knowledge-based true or false statements is shown in Figure 1. The counts and percentages of individuals who responded correctly to the seven knowledge-based true or false statements in the pre-and post-intervention surveys are shown in Table S1.

## 4. Discussion

As concerns grow about the spread of dangerous influenza strains, such as H5N1, and their potential impact on vulnerable REM communities, it is crucial to develop strategies that address barriers to vaccine acceptance [52]. Through our community-based educational intervention, we gained valuable insights into the post-COVID-19 era attitudes of REM individuals regarding the influenza virus and the vaccine. We observed significant improvements in influenza vaccine literacy among REM individuals when comparing pre-and post-intervention survey findings. Additionally, a higher percentage of respondents indicated a high likelihood of receiving the influenza vaccine after the intervention, in contrast to the responses received in the baseline survey.

When examining the attitudes toward the risk of contracting influenza, our study’s results were consistent with findings from previous studies on REM individuals. Most survey participants believed they had little to no risk of contracting the illness. Moreover, many thought that if they did contract the virus, it would have a minimal impact on their health, including the possibility of hospitalization due to pneumonia. This perspective is particularly concerning, as it sharply contradicts the actual risks associated with the virus and its effects on REM communities, especially for Black adults aged 65 and older, who comprised the majority of our study’s participants. A recent study based on pre-pandemic influenza data showed that older patients aged 60 and older were more likely to be hospitalized due to viral infection [53]. More specifically, Black individuals reportedly had the highest age-adjusted hospitalization and intensive care admission (ICU) rates [53]. Vulnerability, including low SES and crowded housing conditions, may contribute to higher hospitalization rates. Another study found that individuals residing in areas with high-poverty census tracts were at a higher risk of severe influenza outcomes [54]. This is an additional troubling fact, as 100% of the individuals included in our study resided in an area of medium-high to high vulnerability. Thus, they were likely at an increased risk of infection despite believing otherwise.

Responses regarding the perceived benefits of vaccination also mirrored those reported in the literature, indicating a general lack of confidence in the vaccine among REM groups. Fewer than 50% of participants believed that the vaccine was “very effective” at preventing the disease, alleviating symptoms, or reducing the risk of complications. Previous studies that explored perceptions of the influenza vaccine among older REM adults showed that many individuals in these groups associate the need for vaccination with being acutely or chronically ill [29,32,48]. This notion of “good health”, combined with the belief that the vaccine is either “moderately effective” or “somewhat effective” at preventing influenza, may contribute to apathetic attitudes toward vaccination.

In addition to perceptions of good health and vaccine benefits, social norms—including vaccine-related attitudes and behaviors of immediate family or close friends—also play a role in influenza vaccine uptake for REM individuals. A prior study survey showed that 36% of Black individuals indicated that their spouse or partner was influential in their vaccine decision, and this was closely followed by their children [32]. In our study, 58% of survey participants believed that “many” of the individuals in the US were vaccinated against influenza each season. Nonetheless, only 45% of the survey participants believed that “many” individuals in their communities were immunized against the virus. These results likely reflect participants’ insights from personal conversations with immediate family members and their broader internal community about the influenza vaccine.

Contrary to previous studies, our survey found that participants did not perceive significant barriers to receiving the vaccine, including concerns about adverse events related to vaccination. Most respondents viewed the vaccine as affordable and convenient to obtain, and 51% indicated they were “unlikely” or “very unlikely” to experience side effects. Notably, 44% of the respondents were over the age of 65, making them eligible to receive vaccinations at no charge through Medicare Part B and Part D [12]. This suggests that the vaccination process is likely both affordable and accessible for this demographic. Furthermore, while concerns about vaccine side effects may have deterred some REM individuals before the COVID-19 pandemic, these concerns may have lessened over time. As global educational efforts regarding respiratory viral vaccinations have progressed, many individuals, especially those aged 65 and older, have received multiple doses since 2021 [7]. Therefore, their real-world experiences with respiratory viral vaccinations may have alleviated previous fears.

Although some perceptions about the influenza virus and vaccine from the pre-intervention survey were concerning, the results of the post-intervention survey demonstrate the significant impact that tailored education from a trusted messenger can have on altering virus-related attitudes. In our study, more than 80% of the individuals who completed the post-intervention survey answered that they trusted the information provided “A great deal”. Previous studies showed that education from a trusted healthcare professional can dissuade negative vaccination-related attitudes among REM individuals. In addition to healthcare providers, religious leaders were also shown to be trusted communicators within REM communities [41,55]. Thus, our strategy of coupling a trusted academician, the FBO, and the lead pastors of the churches in executing the intervention may attest to the positive attitudes regarding the influenza vaccine and virus shown in the post-intervention responses. In general, the presentation was well-received by the post-intervention survey respondents and “extremely effective” at making most participants re-evaluate their own risk for infection and increasing their awareness of the seriousness of influenza and its complications. This shift in perspective was crucial, especially since the pre-intervention survey revealed that participants perceived themselves at low risk for viral infection.

Furthermore, the results from the post-intervention survey indicated that 86% of respondents believed the presentation was “extremely effective” at increasing their general knowledge about the influenza vaccine. This improved vaccine literacy was evident when comparing the selected answers to the seven knowledge-based true or false statements included in both the pre-and post-intervention surveys. We observed a positive change in the selection of correct responses for each question. Notably, when comparing the pre- and post-intervention survey results, there was an 18% difference in correct responses to the statement, “The flu vaccine can cause you to get the flu”. Previous studies showed that more than 30% of Black individuals held the belief that the influenza vaccine could cause viral infection [56]. This misconception was found to contribute to lower vaccination rates among this group [56]. However, our findings suggest that targeted education can significantly change this perception.

Research showed that REM individuals may be unaware of guideline-based vaccination recommendations, particularly older REM adults who often lack knowledge about the stronger influenza vaccines recommended for their age group [29,32,35]. Our tailored educational approach, which focused on these important areas, led to a 30% shift in correct responses to the statement, “The flu vaccine is recommended for everyone, regardless of age or health status”, when comparing the pre- and post-intervention results. Additionally, there was a 40% improvement in the selection of correct responses to the statement, “Stronger versions of the flu vaccine are recommended for older adults (65 years and older)”. This highlights the effectiveness of tailored education in significantly improving influenza vaccine literacy around routine immunizations and the importance of receiving them, even in the absence of acute or chronic illnesses.

Past studies showed that vaccine literacy is a strong predictor of vaccine intention, and our study further supported this finding. In addition to the substantial improvements in the number of correct responses to vaccine knowledge-based statements, we also noted that a higher percentage of post-intervention survey respondents reported a higher likelihood of vaccination following the presentation compared with those who completed the baseline survey (65% vs. 81%). While this improvement in vaccine acceptance could have been due to many factors, receiving a strong recommendation from a trusted healthcare provider was shown to encourage vaccine acceptance among REM individuals [39]. Notably, 82% of the participants in our study expressed trust in the information provided, and more than 65% felt that the presentation effectively addressed their misconceptions or myths about the vaccine. This trust, combined with the alleviation of pre-existing fears or misconceptions, likely contributed to the improved vaccine acceptance observed in the pre-intervention and post-intervention survey results.

Furthermore, more than 90% of respondents in the post-intervention survey indicated they had a high likelihood of recommending the influenza vaccine to a family member or friend. Given the impact of social norms on vaccine acceptance and uptake among REM individuals, it is crucial to empower these individuals to advocate for and recommend the vaccine within their immediate and extended communities [32]. These results showcase that providing access to trustworthy information, disseminated through a trusted healthcare provider, can be transformative for vaccine literacy and acceptance within vulnerable REM communities.

Our study had several strengths that warrant further discussion. To our knowledge, this was one of the first studies to provide insights into the post-COVID-19 pandemic attitudes of vulnerable REM individuals in Southern California regarding the influenza virus and vaccine. An additional strength of this study was the highlighting of the importance of a community–academic partnership in facilitating connections with local vulnerable communities. Furthermore, our research provided valuable insights into how educational interventions can help overcome limitations in influenza vaccine literacy and acceptance observed in vulnerable REM communities.

Despite these strengths, our study also had several limitations. First, because our study was anonymous, we could not link the pre-and post-intervention survey results to conduct parametric statistical testing. We also could not test for statistical significance regarding the shift in vaccine acceptance due to the different rating scales applied in the pre-and post-intervention surveys. Furthermore, this study only captured immediate post-intervention effects, and due to the anonymity, we were unable to assess long-term knowledge retention. Although we held a vaccine clinic immediately after the intervention (on the same day and at the same site) where vaccinations were provided, we were unable to confirm the vaccination status of the survey respondents due to the anonymity of the questionnaire. Because of these factors and the absence of a control comparator group, the intervention cannot be attributed as the cause of vaccine uptake among any of the participants. Additionally, Black individuals and those who identified as female were the most represented among the survey respondents. As a result, the attitudes and effects of the educational intervention may vary in other vulnerable demographic groups. This study was also conducted in Southern California, primarily involving the church’s congregants as participants. Therefore, the results may not accurately reflect the perspectives of other vulnerable groups nationwide. The Likert scale used to assess attitudes may have introduced bias, as the respondents might have agreed with statements they did not fully understand. Moreover, the phrasing of the post-intervention survey questions and the use of Likert scales may have also led to a social desirability bias. Additionally, the USD 20 gift card incentive could have influenced participation and responses, potentially introducing a selection and response bias. Furthermore, the lack of an “I do not know” option for the true or false knowledge-based statements may have resulted in skewed findings, with individuals feeling pressured to select either “true” or “false” when a more suitable response was unavailable.

## 5. Conclusions

Disparities in the acceptance and uptake of vaccines have significantly increased since the COVID-19 pandemic. This trend is likely to result in adverse health outcomes for the most vulnerable populations. By utilizing a community-based educational intervention, we effectively communicated the risks associated with the influenza virus and dispelled myths that often hinder vaccination among vulnerable REM individuals. As a result, we observed a notable improvement in vaccine literacy and acceptance among the REM participants involved in the intervention. However, further research is required to address vaccination challenges in the post-COVID-19 era.

## Figures and Tables

**Table 1:** Demographics of baseline participants.

Variable	Pre-Survey Participants (n = 116) (%)
**Race/Ethnicity**	
Non-Hispanic Black or African American	99 (85%)
American Indian or Alaskan Native	1 (1%)
Two or more races	4 (3%)
Hispanic/Latino(a)	9 (8%)
Other	3 (3%)
**Age**	
18–24	3 (3%)
25–34	6 (6%)
35–44	7 (7%)
45–54	13 (11%)
55–64	25 (22%)
65 years or older	51 (44%)
**Gender**	
Male	46 (40%)
Female	70 (60%)
**Level of Social Vulnerability**	
High	115 (99%)
Medium-high	1 (0.9%)

Shown in Table 1 are the demographics (race/ethnicity, age, and gender) collected from the individuals who completed the pre-intervention survey. Zip codes were also collected from the individuals who completed the baseline survey. The zip codes were inputted into the CDC SVI tool to determine the level of social vulnerability.

**Table 2:** Pre-intervention survey responses on the perceptions of the influenza virus and vaccine and intent to be vaccinated.

Area of Focus	Variables	Number of Respondents to Each Likert Ranking (%) (n = 116)					
**Perceived risk of Influenza Infection**	How likely are you to get the flu this season?	Very unlikely	Unlikely	Likely	Very likely	No response	
		16 (14%)	47 (41%)	32 (28%)	17 (15%)	4 (2%)	
	How severe do you think your flu symptoms would be if you were to get it this season?	Negligible	Mild	Significant	Intense	No response	
		10 (9%)	75 (65%)	17 (15%)	8 (7%)	6 (5%)	
	How much do you worry about being hospitalized or developing pneumonia if you were to get the flu this season?	Not worried at all	Slightly worried	Moderately worried	Very worried	No response	
		64 (55%)	25 (22%)	19 (16%)	5 (4%)	3 (3%)	
**Perceived Influenza Vaccine Benefits**	How effective do you believe the influenza vaccine is (Stem question):	Not Effective	Somewhat Effective	Moderately Effective	Very Effective	No response	
	in preventing you from catching the flu?	8 (7%)	22 (19%)	43 (37%)	37 (32%)	6 (5%)	
	in reducing the severity of symptoms if you get infected?	4 (3%)	27 (23%)	32 (28%)	48 (41%)	5 (4%)	
	in reducing your risk of complications like hospitalization or pneumonia?	3 (3%)	22 (19%)	32 (28%)	54 (47%)	5 (4%)	
**Perceived Barriers in Receiving the Influenza Vaccine (including Accessibility barriers)**	How affordable do you find the influenza vaccine?	Not affordable	Somewhat affordable	Moderately affordable	Very affordable	No response	
		3 (3%)	18 (16%)	9 (8%)	77 (66%)	9 (8%)	
	How convenient do you find the process of getting the influenza vaccine?	Not convenient	Somewhat convenient	Moderately convenient	Very convenient	No response	
		4 (3%)	11 (9%)	13 (11%)	82 (71%)	6 (5%)	
	How likely are you to experience side effects from the flu vaccine?	Very unlikely	Unlikely	Likely	Very likely	No response	
		14 (12%)	45 (39%)	32 (28%)	20 (17%)	5 (4%)	
**Perceived Community Influenza Vaccine Uptake**	How many people in the US do you think get a flu vaccine every year?	Very few	Some	Many	Nearly everyone	No response	
		10 (10%)	33 (26%)	65 (58%)	5 (5%)	1 (1%)	
	How many people in your community do you think get the flu vaccine every year?	6 (5%)	50 (43%)	52 (45%)	2 (2%)	6 (5%)	
**Intent to be Vaccinated (Vaccine Acceptance-Related)**	How likely are you to get the flu vaccine if there is a convenient and easily accessible location for vaccination?	Very Unlikely	Unlikely	Likely	Very Likely	Extremely Likely	No response
		25 (22%)	14 (12%)	32 (28%)	30 (26%)	13 (11%)	2 (2%)

Shown in Table 2 are the responses (represented through counts and percentages) to the pre-intervention survey questions on the participants’ perceptions of the influenza virus and vaccine and their intent to be vaccinated. The number of individuals that responded with “likely”, “very likely”, and “extremely likely” were combined to represent a high likelihood of receiving the vaccine. “Flu” was used instead of influenza to prioritize the use of plain language throughout the survey.

**Table 3:** Post-intervention survey responses on the impact of the intervention on influenza virus and vaccine perceptions and intent to be vaccinated.

**Variable**	**Average Score (1–5 Scoring) (1 = “Not Effective”, 5 = “Extremely Effective”)** **(n = 90)**	**Number Responding with “5” Indicating an “Extremely Effective” Response (%) (n = 90)**	
How effective was the presentation in increasing your general knowledge about the flu vaccine	4.84	77 (86%)	
How effective was the presentation in helping you trust the safety of the flu vaccine	4.77	66 (73%)	
How effective was the presentation in helping you understand the benefits of the flu vaccine	4.85	77 (85%)	
How effective was the presentation in addressing any misconceptions or myths about flu vaccines that you may have had	4.67	59 (65%)	
How effective was the presentation in making you re-evaluate your own risk of getting the flu this season	4.65	54 (60%)	
How effective was the presentation in making you aware of the seriousness of the flu and its complication	4.82	73 (81%)	
How much do you trust the information provided in the presentation about flu vaccines?	**Average Score (1–5 Scoring) ** **(1 = “Not at all”, 5 = “A great deal”)** **(n = 90)**	**Number Responding with “5”, Indicating “A great deal” Response (%) ** **(n = 90)**	
	4.87	74 (82%)	
**Vaccine-Acceptance-Related Questions**			
How important do you feel it is to get the flu vaccine?	**Average Score** **(1–5 Scoring)** **(1 = “Not important”, 5 = “Extremely Important”)** **(n = 90)**	**Number Responding with “5”, Indicating an “Extremely Important” Response** **(n = 90)**	
	4.6	74 (82%)	
How likely are you to recommend the flu vaccine to a family member or friend?	**Average Score** **(1–5 Scoring)** **(1 = “Extremely Unlikey”, 5 = “Extremely Likely”)** **(n = 90)**	**Number Responding with a “4” or “5”, Indicating “High Likelihood”** **(n = 90)**	**Number Responding with “5”, Indicating an “Extremely Likely” Response** **(n = 90)**
	4.7	84 (93%)	69 (77%)
How likely are you to get the flu vaccine after the presentation? (intent to be vaccinated)	**Average Score** **(1–5 Scoring)** **(1 = “Extremely Unlikey”, 5 = “Extremely Likely”)** **(n = 90)**	**Number Responding with a “4” or “5”, Indicating “High Likelihood”** **(n = 90)**	**Number Responding with “5”, Indicating an “Extremely Likely” Response** **(n = 90)**
	4.5	73 (81%)	65 (72%)

Shown in Table 3 are the responses to the post-intervention survey questions (shown by counts and percentages) assessing the impact of the intervention on the attitudes of the participants. Scoring was achieved via a selection of a score of 1–5 on a Likert scale, and the mean score for each selection is shown in this table. We also include the percentage of the individuals that responded with a “5”, the highest score awarded. For the question that measured the intent to be vaccinated and to recommend the vaccine to others, we also include the counts and percentages of those that responded with a “4” or “5”, indicating a high likelihood. “Flu” was used instead of influenza to prioritize the use of plain language throughout the survey.

## Data Availability

The original contributions presented in this study are included in this article/Supplementary Materials. Further inquiries can be directed to the corresponding author.

## Supplementary Materials

The following supporting information can be downloaded from https://www.mdpi.com/article/10.3390/idr17020018/s1 Document S1: Influenza study pre-intervention survey, Supplemental Document S2: Influenza study post-intervention survey, Table S1: Counts and percentage of correct responses to the knowledge-based true or false statements (pre-intervention survey vs. post-intervention survey) (measurement of vaccine literacy shift).
