# Supplementary material for: Utilizing an Educational Intervention to Enhance Influenza Vaccine Literacy and Acceptance Among Minoritized Adults in Southern Californian Vulnerable Communities in the Post-COVID-19 Era
Source: Infect Dis Rep. 2025 Feb 26;17(2):18. doi: 10.3390/idr17020018 (PMC11932246; doi:10.3390/idr17020018)
Supplement: Supplementary file 1 [file idr-17-00018-s001.zip › Supplemental Document 1.pdf]

Supplemental Document 1: Influenza Study Pre-Intervention Survey

Thank you for taking part in this important survey. Before we start our presentation, we would like to learn more about your thoughts on flu vaccines. Completing this survey will only take a few minutes of your time. Your answers will help us find better ways to address any concerns or questions people may have about flu shots.

First, we'd like to ask a few quick questions about you. This information will help us understand the people participating in our survey and make sure our findings represent a diverse group.

1. **Please provide your zip code: ________________________**

2. **What is your current gender?**
   a. Man
   b. Woman
   c. Nonbinary
   d. Other

3. **What is your age?**
   a. 18-24 years old
   b. 25-34 years old
   c. 35-44 years old
   d. 45-54 years old
   e. 55-64 years old
   f. 65 years old or above

4. **Are you of Hispanic, Latino/a, or Spanish origin?**
   a. Yes
   b. No

5. **Which categories describe you?**
   a. American Indian or Alaska Native
   b. Asian
   c. Black or African American
   d. Native Hawaiian/Other Pacific Islander
   e. White
   f. Two or More Races
   g. Some Other Race

Section 1:

As you consider the current flu season, please answer a few questions about your worries or lack of concerns regarding it.

6. **How likely are you to get the flu this season?**
    a. Very unlikely
    b. Unlikely
    c. Likely
    d. Very likely

7. **How severe do you think your flu symptoms would be if you were to get it this season?**
    a. Negligible
    b. Mild
    c. Significant
    d. Intense

8. **How much do you worry about being hospitalized or developing pneumonia if you were to get the flu this season?**
    a. Not worried at all
    b. Slightly worried
    c. Moderately worried
    d. Very worried

Now, let's talk about any potential challenges you might face when considering the flu vaccine. Please answer the following questions:

9. **How affordable do you find the flu vaccine?**
    a. Not affordable
    b. Somewhat affordable
    c. Moderately affordable
    d. Very affordable

10. **How convenient do you find the process of getting the flu vaccine?**
    a. Not convenient
    b. Somewhat convenient
    c. Moderately convenient
    d. Very convenient

11. **How likely do you think you are to experience side effects from the flu vaccine?**
    a. Very unlikely
    b. Unlikely
    c. Likely

d. Very likely

Let's look into the positive effects of getting the flu vaccine. Please answer these questions about how well you think the flu vaccine works.

How effective do you believe the flu vaccine is in:

12. **Preventing you from catching the flu?**
    a. Not effective
    b. Somewhat effective
    c. Moderately effective
    d. Very effective

13. **Reducing the severity of symptoms if you get infected?**
    a. Not effective
    b. Somewhat effective
    c. Moderately effective
    d. Very effective

14. **Reducing your risk of complications like hospitalization or pneumonia?**
    a. Not effective
    b. Somewhat effective
    c. Moderately effective
    d. Very effective

Let's now explore what people in your community think about getting the flu shot. Please answer these questions about how interested the community is in getting the flu vaccine.

15. **How many people in the US do you think get a flu vaccine every year?**
    a. Very few
    b. Some
    c. Many
    d. Nearly everyone

16. **How many people in your community do you think get the flu vaccine every year?**
    a. Very few
    b. Some
    c. Many
    d. Nearly everyone

Section 2:

The following questions contain statements people make about flu vaccines. Please determine whether these statements are accurate or inaccurate.

1. **The flu vaccine boosts your body's natural immune response.**
    a. True
    b. False

2. **The flu vaccine can still lessen the severity and duration of flu symptoms, even if it doesn't cover all types of viruses going around.**
    a. True
    b. False

3. **The flu vaccine this year is less effective than in most years.**
    a. True
    b. False

4. **The flu vaccine is recommended for everyone, regardless of age or health status.**
    a. True
    b. False

5. **The flu vaccine can cause you to get the flu.**
    a. True
    b. False

6. **The flu vaccine is unnecessary if you haven't had the flu in several years.**
    a. True
    b. False

7. **Stronger versions of the flu vaccine are recommended for older (65 year+) adults.**
    a. True
    b. False

Section 3:

1. **How likely are you to get the flu vaccine if there is a convenient and easily accessible location for vaccination?**
    a. Extremely unlikely (1)
    b. Unlikely (2)
    c. Likely (3)
    d. Very likely (4)
    e. Extremely likely (5)
