# Supplementary material for: Utilizing an Educational Intervention to Enhance Influenza Vaccine Literacy and Acceptance Among Minoritized Adults in Southern Californian Vulnerable Communities in the Post-COVID-19 Era
Source: Infect Dis Rep. 2025 Feb 26;17(2):18. doi: 10.3390/idr17020018 (PMC11932246; doi:10.3390/idr17020018)
Supplement: Supplementary file 1 [file idr-17-00018-s001.zip › Supplemental document 2.pdf]

Supplementary Document 2: Influenza Study Post-Intervention Survey

Thank you for coming to our presentation on the flu vaccination. We hope you found it informative and enjoyable. Please take a few moments to answer the following questions. Your feedback is valuable to us!

Section 1:

On a scale of 1 to 5, how effective was today's presentation:

1. **in increasing your general knowledge about the flu vaccine?**

   Not at all effective 1 2 3 4 5 Extremely Effective

2. **in helping you understand the benefits of the flu vaccine?**

   Not at all effective 1 2 3 4 5 Extremely Effective

3. **in helping you trust the safety of the flu vaccine?**

   Not at all effective 1 2 3 4 5 Extremely Effective

4. **in addressing any misconceptions or myths about flu vaccines that you may have had?**

   Not at all effective 1 2 3 4 5 Extremely Effective

5. **in making you re-evaluate your own risk of getting the flu this season?**

   Not at all effective 1 2 3 4 5 Extremely Effective

6. **in making you aware of the seriousness of the flu and its complications?**

   Not at all effective 1 2 3 4 5 Extremely Effective

7. **How much do you trust the information provided in the presentation about flu vaccines?**

   Not at all 1 2 3 4 5 A great deal

8. **How important do you believe it is for you to get the flu vaccine?**

   Not at all Important 1 2 3 4 5 Extremely Important

Section 2:

1. **The flu vaccine boosts your body's natural immune response.**
   a. True

   b. False

2. **The flu vaccine can still lessen the severity and duration of flu symptoms, even if it doesn't cover all types of viruses going around.**
   a. True
   b. False

3. **The flu vaccine this year is less effective than in most years.**
   a. True
   b. False

4. **The flu vaccine is recommended for everyone, regardless of age or health status.**
   a. True
   b. False

5. **The flu vaccine can cause you to get the flu.**
   a. True
   b. False

6. **The flu vaccine is unnecessary if you haven't had the flu in several years.**
   a. True
   b. False

7. **Stronger versions of the flu vaccine are recommended for older (65 year+) adults.**
   a. True
   b. False

Section 3:

1. **How likely are you to get a flu vaccine after the presentation?**

   Extremely unlikely 1 2 3 4 5 Extremely Likely

2. **How likely are you to recommend the flu vaccine to a family member or friend?**

   Extremely unlikely 1 2 3 4 5 Extremely Likely

Any other comments or feedback: ____

____
