# Supplementary material for: Utilizing an Educational Intervention to Enhance Influenza Vaccine Literacy and Acceptance Among Minoritized Adults in Southern Californian Vulnerable Communities in the Post-COVID-19 Era
Source: Infect Dis Rep. 2025 Feb 26;17(2):18. doi: 10.3390/idr17020018 (PMC11932246; doi:10.3390/idr17020018)
Supplement: Supplementary file 1 [file idr-17-00018-s001.zip › Table S1.pdf]

Table S1. Counts and Percentages of Correct Responses to the Knowledge-based True or False Statements (Pre-Intervention Survey vs. Post-Intervention Survey (Measurement of Vaccine Literacy Shift)

**True or False Statement.**	**Respondents that answered correctly in the Pre-Intervention Survey (n=116) (%)**	**Respondents that answered correctly Post-Intervention Survey (n=90) (%)**	**P-value**
The flu vaccine boosts your body's natural immune response.	87 (75%)	85 (94%)	$p<0.05$
The flu vaccine can still lessen the severity and duration of flu symptoms, even if it doesn't cover all types of viruses going around.	98 (84%)	86 (96%)	$p<0.05$
The flu vaccine this year is less effective than in most years.	88 (76%)	79 (88%)	$p<0.05$
The flu vaccine can cause you to get the flu.	67 (58%)	68 (76%)	$p<0.05$
The flu vaccine is unnecessary if you haven’t had the flu in several years.	95 (82%)	82 (91%)	$p>0.05$ ($p=0.06$)
The flu vaccine is recommended for everyone, regardless of age or health status.	77 (57%)	78 (87%)	$p<0.05$
Stronger versions of the flu vaccine are recommended for older (65 year+) adults.	54 (47%)	78 (87%)	$p<0.05$

Shown in **Table S1** is a comparison of the counts and percentages of correct responses to the seven true or false influenza knowledge-based statements included in the pre- and post-intervention surveys. “Flu” was used instead of influenza to prioritize the use of plain language throughout the survey. ($p<0.05$) denotes a statistically significant difference.
